# The Involvement of Glycosaminoglycans in Airway Disease Associated with Cystic Fibrosis

**DOI:** 10.1100/tsw.2011.81

**Published:** 2011-04-19

**Authors:** Emer P. Reeves, David A. Bergin, Michelle A. Murray, Noel G. McElvaney

**Affiliations:** Respiratory Research Division, Department of Medicine, Royal College of Surgeons in Ireland, Education and Research Centre, Beaumont Hospital, Dublin, Ireland

**Keywords:** cystic fibrosis, glycosaminoglycans, airway inflammatory disease, neutrophil antimicrobial peptides and proteases

## Abstract

Individuals with cystic fibrosis (CF) present with severe airway destruction and extensive bronchiectasis. It has been assumed that these structural airway changes have occurred secondary to infection and inflammation, but recent studies suggest that glycosaminoglycan (GAG) remodelling may be an important independent parallel process. Evidence is accumulating that not only the concentration, but also sulphation of GAGs is markedly increased in CF bronchial cells and tissues. Increased expression of GAGs and, in particular, heparan sulphate, has been linked to a sustained inflammatory response and neutrophil recruitment to the CF airways. This present review discusses the biological role of GAGs in the lung, as well as their involvement in CF respiratory disease, and their potential as therapeutic targets.

## INTRODUCTION

Cystic fibrosis (CF) is an autosomal-recessive disorder caused by mutation of the CF transmembrane regulator (CFTR), which functions as a chloride channel on epithelial cell membranes. CF affects 1/2500 births in the Caucasian population. There are over a 1000 CFTR mutations that cause disease in many organs, including lungs, pancreas, liver, sinuses, bowel, and reproductive organs. Almost all people with CF develop chronic airway disease related to airway obstruction, inflammation, and recurrent infections with various pathogens, most commonly *Pseudomonas aeruginosa* (*P. aeruginosa*) and *Staphylococcus aureus* (*S. aureus*). Airway disease in CF is further characterised by tenacious mucoid secretions and excessive neutrophil recruitment into the lungs, the latter playing a pivotal role in the destruction of lung tissue by release of proteolytic enzymes that overwhelm the antiprotease defences of the lung[1]. Indeed, a strong debate continues among clinicians and scientists on whether inflammation or infection occurs first in children with CF[2,3,4]. For example, studies investigating early pulmonary inflammation in infants with CF showed increased interleukin (IL)-8 and neutrophil burden in the absence of bacterial colonisation[2,5], suggesting dysregulation of the inflammatory response within the CF lung. Transgenic animals have been employed to resolve this debate and, in contrast to the mouse model of CF that shows little airway pathology[6], studies with ferret or pig *CFTR*-knockout models illustrate that CF-related reductions in gland fluid secretions resemble those seen in human CF[7,8]. Studies employing the CFTR pig model observed spontaneous development of characteristics related to CF lung disease, including impaired ability to eradicate bacteria and airway inflammation within months of birth[9]. The results of this study concluded that impaired bacterial clearance is the pathogenic event that initiates inflammation in the CF lung. In contrast, however, studies expressing inflammation as a function of bacterial quantity revealed that the bronchoalveolar lavage fluid (BALF) of children with CF contained significantly increased levels of IL-8 and neutrophil numbers compared to children with other chronic respiratory conditions[10], and the data presented argued against higher levels of bacterial numbers or endotoxin as a cause of increased inflammation[11]. Moreover, our understanding of the pathophysiology of CF is currently undergoing a significant reassessment and several lines of evidence have converged, highlighting the importance of glycosaminoglycans (GAGs) in CF airway inflammatory disease. Understanding the structure of GAGs has been facilitated by the discovery of GAG synthases, specific degrading enzymes, and the study of specific carbohydrate arrays[12]. In the lung, GAGs are distributed in the extracellular matrix (ECM), encompassing the interstitial space lying between the capillary endothelium and the alveolar epithelium[13,14] (Fig. 1), in the subepithelial tissue, bronchial walls, and airway secretions. In addition to playing key structural roles, GAGs participate in a number of physiologic processes and are involved in cell migration and differentiation, antigen recognition, cell adhesion and communication, and wound healing. For example, the interaction of inflammatory molecules with GAGs is thought to provide a mechanism for establishing chemokine gradients in order to provide directional signals for migrating cells[15]. Cytokine- and chemokine-GAG interactions are also involved in the presentation of inflammatory molecules to respective receptors and protection from proteolytic degradation. Examples of cytokines bound to GAGs in a steady state include binding of heparan sulphate (HS) to interferon-gamma (IFN-γ)[16]□, limiting the extent of its carboxyl-terminal domain degradation[17], yet decreasing the plasma clearance and increasing the cytokine activity by up to 600%[18]. *In vitro* studies have also shown that binding of secreted proteases, including kallikrein to hyaluronic acid (HA), reduced catalytic activity[19], whilst soluble HA increased ciliary beat frequency of tracheal epithelial cells[20].

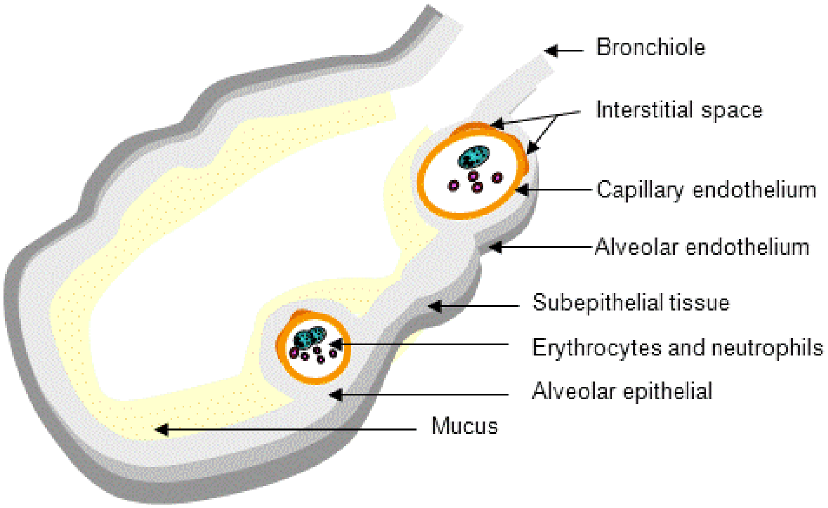

**FIGURE 1.** Schematic representation of a lung alveolus. GAGs are distributed in the interstitial space between the capillary endothelium and the alveolar epithelium, in the subepithelial tissue, and airway mucus.

GAGs are long, linear structures composed of heterogeneous polysaccharides and are formed by long disaccharide units with varying degrees of linkage, acetylation, and sulphation (Fig. 2). The disaccharide units include galactose, *N*-acetylglucosamine, *N*-acetylgalactosamine, and glucuronic acid. The chain lengths can vary hugely and the molecular weight can reach up to three orders of magnitude, thus allowing these chains to have great variance in structure and size. GAGs are divided into two principal types: nonsulphated, which consist of HA, and sulphated, which include dermatan sulphate (DS), keratin sulphate (KS), HS, heparin, and chondroitin sulphate (CS). Significant changes occur in concentration and composition of GAGs at sites of inflammation. For example, a reduced level of HS and increased CS have been observed in atherosclerosis[21]. Increased concentrations of GAGs[22,23] have been found in BALF from children with CF, and secretion of HS[24], CS[25,26], and HA[27,28] is markedly increased in bronchial cells and CF tissues. This review will present some of the important insights into GAG biology, and will consolidate the literature in order to discuss the prospective role of GAGs in CF and their potential as therapeutic targets.

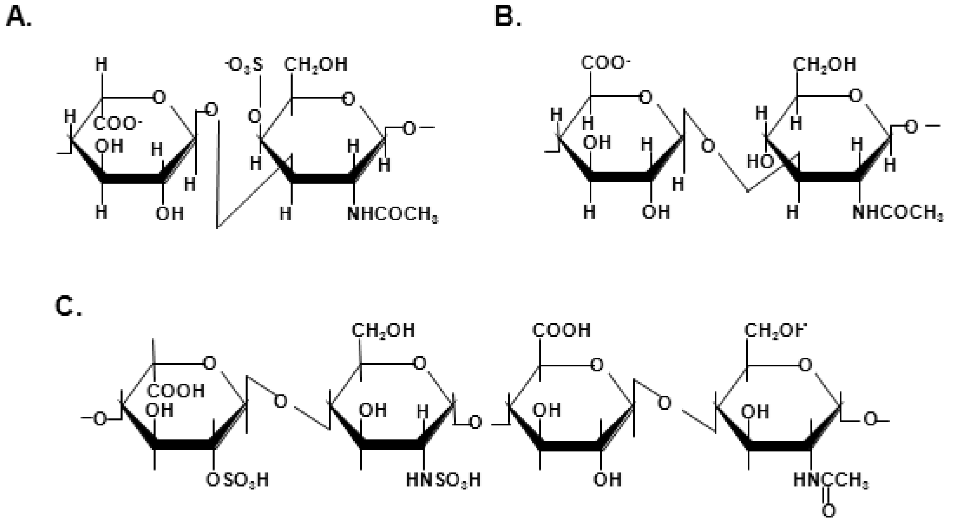

**FIGURE 2.** Structural formulas of GAGs up-regulated in CF. Structures represent the repeating units of chondroitin sulphate B (A), HA (B), and HS (C).

## DYSREGULATED HYALURONIC ACID, HEPARAN SULPHATE, AND CHONDROITIN SULPHATE EXPRESSION IN THE CF AIRWAYS

HA, also known as hyaluronan, located in peribronchial and perialveloar tissue[29] and within the lung, has a molecular weight of 220 kD[30]. HA is made by mesenchymal cells and is the only major extracellular oligosaccharide that is not covalently linked to protein. It is composed of up to 50,000 repeats of disaccharide units composed of glucuronic acid and *N*-acetylglucosamine. Due to the large number of hydrophilic residues on its surface, HA binds water molecules and cations via its $COO^-$ groups, thus increasing both ion and osmotic pressure within the HA gel[31]. HA therefore plays an important role in the hydration of tissues, and structurally is an integral part of elastin fibres and the microfibrils of collagen[32,33]. HA functioning in healthy tissue and remodelling associated with disease also relies upon interaction with HA-binding proteins and receptors, termed hyalderins[34]. These include the cell surface receptor CD44 and RHAMM (receptor for HA-mediated motility). The binding of HA to CD44 is involved in leukocyte rolling, activation, tumour metastasis, and healing lung inflammation[35]. HA binding to RHAMM is important in cell migration, proliferation, and motility[36]. A recent study by Jiang and colleagues has demonstrated the importance of HA signalling via TLR2 and TLR4 for initiation of an inflammatory response to lung injury[37]. *In vivo* studies performed in murine *Myd88*$^{-/-}$ and *Tlr4*$^{-/-}$ *Tlr2*$^{-/-}$ knockout models or inhibition of HA binding resulted in reduced inflammatory response, increased epithelial cell apoptosis, and decreased transmigration of neutrophils[37]. In contrast, targeted deletion of TLR1, TLR3, TLR5, or TLR9 had no effect on HA-induced chemokine expression. The HA receptor complex involved in noninfectious inflammation has been shown to involve TLR4, MD-2, and CD44, distinct from the TLR4, MD-2, and CD14 complex that recognises lipopolysaccharide (LPS) during infection[38] (Fig. 3). However, additional studies have shown that HA can stimulate chemokine production independent of CD44 and that CD44 regulation of HA signalling may in fact be cell-type dependent[37,39,40].

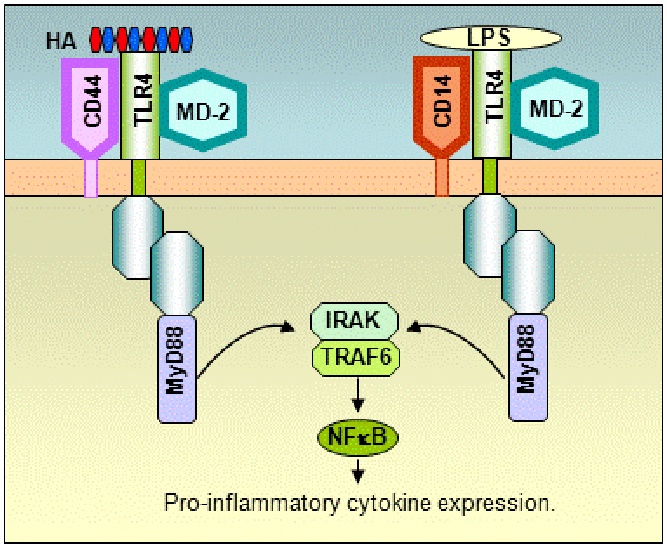

**FIGURE 3.** Comparable TLR4 signalling of HA and LPS. Adapted from Jiang et al.[129].

The presence of small-molecular-weight HA fragments can arise due to changes in expression or enzymatic activity of enzymes involved in its synthesis and catabolism, including HA synthases (HAS)-1, HAS-2, and HAS-3. Whilst HAS-1 and -2 are involved in synthesis of high-molecular-weight HA, HAS-3 produces shorter HA fragments[41]. Several studies have shown disparate biological effects of HA depending on molecular mass. For example, low-molecular-weight HA fragments can stimulate mouse macrophages to express chemokines and cytokines, and enhance IFN-γ–induced MIG (monokine induced by gamma) expression through the NF-κB pathway[42]. In contrast, however, aerosolisation of high-molecular-weight HA in animal studies decreased serine protease–induced alveolar injury[43] and has been administered to human subjects with no reported adverse effects[44,45]. In CF, significantly increased serum[28] and bronchial levels of HA have been reported[46]. The HA concentration in CF sputum ranged from 124 to 784 µg/g and was 100-fold higher than in acute bronchitis. Moreover, it was shown that CFTR functions to transport HA across membranes of epithelial cells and that the described transport is defective in patients with CF[46]. Within this latter study, the authors suggest that the intracellular accumulation of HA in CF epithelial cells may lead to the reported swelling and oncosis that is caused by defective anion channels[47].

HS is an abundant acidic GAG that is expressed on almost all cells in the body. HS is a major component of the ECM and by binding laminin supports basement membrane structure[48]. In addition, the interaction between HS and collagen type V plays a significant role in modulation of cell-substratum adhesion[49]. The structure of HS is complex, and HS chains are polymerised by the sequential addition of β-D-glucuronic acid and *N*-acetyl-α-D-glucosamine (GlcNAc) monosaccarides. GlcNAc residues can be modified by N-deacetylase/*N*-sulphotransferase enzymes and by *O*-sulphation at different sites by sulphotransferase enzymes. Due to the strong charge of HS, it interacts noncovalently with basic proteins and is involved in cell-matrix interactions, activation of cytokines, enzymes and growth factors[50]. HS reversibly interacts with cytokines such as IL-5, IL-6, IL-8, IL-10, tumour necrosis factor-alpha (TNF-α), and platelet factor 4[51,52,53]. HS binding to various proteins can also determine its action. For example, *in vitro* studies have illustrated that HS moieties of proteoglycans, including syndecan-1, act as consensus binding sites for IL-8[54,55]. Binding to GAGs renders IL-8 impervious to proteolysis, thus increasing the half-life and activity of the chemokine at the site of inflammation within the airways[24,56]. Amino acids involved in IL-8:GAG binding have been identified as the basic amino acids, His-18, Arg-60, Lys-64, Lys-67, and Arg68, located in the C-terminal helix, and Lys20 in the proximal loop[57]. In addition, prevention of binding between cell surface HS and basic fibroblast growth factor substantially reduces binding of the growth factor to its cell-surface receptor[58]. In CF, increased expression of HS and subsequent binding to IL-8 has been linked to the sustained inflammatory response and continued recruitment of neutrophils in CF bronchial tissue[24]. HS was found to be significantly more abundant in CF epithelial and endothelial basement membranes compared to samples from patients with chronic obstructive pulmonary disease. The authors concluded that sustained neutrophil recruitment in the CF airway may therefore be related not only to increased IL-8 expression, but also to the increased stability and prolonged activity and retention of IL-8 when it is bound to elevated levels of HS in CF bronchial tissue. Moreover, the potential of IL-8 to determine the fate of other inflammatory molecules, such as IL-18, within the inflammatory milieu of the CF lung has been shown[59]. GAGs are capable of binding IL-18 and, whilst bound, the cytokine is impervious to proteolytic degradation by neutrophil elastase (NE)[60], which is present at high concentrations within the CF lung[61]. The stability of IL-18 is, however, provisional and undermined in the presence of IL-8, which competes and displaces IL-18 from its GAG binding site[59]. In addition, elevated levels of HS within the CF airways may also impact upon microbial colonisation, as HS has been suggested as a cell receptor for a number of pathogenic microorganisms, including adherence of *P. aeruginosa*[62].

CS is composed of linear repeating units (between 15 and 150 units) containing *D*-galactosamine and *D*-glucuronic acid. Several studies have shown the importance of CS GAGs on cell and ECM proteoglycans in the binding of a variety of cell signalling molecules (e.g., growth and differentiation factors, cytokines, chemokines, and enzymes)[63,64,65]. Significantly, it is the presence of specific sulphated saccharide motifs within the CS chain that allows the binding and regulation of many of these signalling molecules. Within a study designed to compare glycoconjugate secretion by intrahepatic biliary epithelial cells from normal livers and livers of patients with CF, markedly increased secretion of CS was quantified in CF biliary epithelium *in vitro* compared with non-CF cells[22]. Relevant to the airways, not only does CF sputum contain significantly elevated levels of CS[26], but experimental studies have shown that mutations to CFTR give rise to aberrant levels of sulphation. Within the respiratory tract, elevated levels of oversulphated GAGs with altered ionic characteristics due to increased sulphate content could contribute to abnormal secretions such as occurs in CF. Data supporting this phenomenon have demonstrated synthesis of oversulphated glycoconjugates by CF tissue in organ culture[66] and airway epithelial cells[67,68]. Within a murine model of CF, the CFTR-mediated effects on sulphation of glycoconjugates were observed to alter ECM structure[69]. Research within this area extends as far back as 1976, when Boat et al. recorded increased sulphation in nonpurulent tracheobronchial secretions of patients with CF compared to patients with chronic bronchitis[70]. Of interest, deficiencies of the enzyme arylsulphatase B (ASB), which catalyses hydrolysis of the sulphate ester of *N*-acetylgalactosamine 4-sulphate of CS, was demonstrated in a lymphoid cell line from CF patients before the genetic defect in CF was described[71,72]. ASB silencing in bronchial epithelial cells led to an accumulation of total cellular sulphated GAGs, including chondroitin-4 sulphate (C4S) and retention of IL-8 by bronchial epithelial cells. The authors concluded that C4S-IL-8 interactions may participate within the altered inflammatory responses of the CF airways[73]. In addition, correction of the genetic defect by CFTR transfection in bronchial epithelial cells significantly increased ASB activity by 40%[74].

Of relevance to the CF lung milieu, certain GAG-protein interactions, including selenoprotein P[75] and platelet endothelial cell adhesion molecule 1 (PECAM-1)[76], are regulated by pH and alterations of the pH can have an effect on the ability of proteins to bind to GAGs. Within CF, pH is a significant parameter to consider, as the pH in airways of individuals with CF is known to be low (pH 5.8–6.6)[77,78]. Results of a study performed by our group revealed increased interaction of IL-18 to GAGs at lower pH, possibly by increasing histidine protonation and, in doing so, favouring the formation of electrostatic interactions with the negatively charged groups of GAGs. Collectively, the described reports indicate that increased expression of sulphated GAGs at the endothelium in CF and reduced pH within the airways may point to an exceptional characteristic of CF that contributes to increased stability of cytokines and chemokines, thus leading to chronic neutrophil infiltration and inflammation[24,56].

## THE EFFECT OF GAGS ON PROTEASE ACTIVITY WITHIN THE CF AIRWAYS

The CF lung environment is associated with a disproportionate inflammatory response and one aspect of this complex inflammatory environment is the high protease burden. NE is a serine protease that is found in high abundance in CF BALF[79,80] and is capable of degrading major components of connective tissue, including elastin[61,81,82,83]. It has been suggested that NE is the most important protease in the inflammatory lung and is placed at the apex of the protease hierarchy[84]. Several studies have shown a direct relationship between sputum NE and disease severity in CF, with highest levels of enzyme being observed in patients with severe lung disease. One of the therapeutic goals in CF management is offsetting this protease-antiprotease imbalance. Some of the earlier aerosol studies using either recombinant secretory leukocyte protease inhibitor (SLPI)[85] or alpha-1 antitrypsin (AAT)[1] highlighted the potential of antiprotease treatment in CF. Studies that have re-examined the effect of aerosolised therapy revealed that recombinant AAT had little impact on inflammation[86], yet in contrast, aerosolised plasma-purified AAT significantly decreased key inflammatory mediators (IL-8, TNF-α, leukotriene B4, and IL-1β), elastase activity, and incidence of *Pseudomonas* infection[87]. In addition, a large number of *in vitro*[88,89,90] and *in vivo*[89,91,92] studies have been performed looking at the antiprotease effects of heparin. The theory behind using heparin to bind and inactivate NE is not dissimilar to what happens naturally *in vivo*. By way of explanation, exposure of HS proteoglycans to NE causes release of HS chains and fragments of HS proteoglycans[93,94], which in turn bind and limit NE activity[95]. However, under chronic inflammatory conditions and high NE burden, this natural feedback loop is destroyed, tilting the balance in favour of NE-mediated tissue destruction. Walsh et al. demonstrated that heparin and HS have the ability to inhibit NE breakdown of lung elastin, thus suggesting a role of these GAGs in protecting structural proteins[96]. This inhibition of NE activity by heparin was initially deemed to be influenced by charge interactions, where correlations were observed between charge density and *N*-sulphate groups on the heparin molecules and NE activity[97]. However, more recent studies suggest that heparin binds to NE by a competitive tight binding mechanism corresponding to length of saccharides, with 12–14 being required for NE inhibition[95]. In addition, semi-synthetic or GAG derivatives have also demonstrated antiprotease activity against NE[98,99] and cathepsin G[99,100]. Moreover, a role for GAGs in stabilising interaction of SLPI with NE has been reported[101], augmenting SLPI antiprotease activity *in vivo*[102].

Other key proteases and peptides that are found in the CF lung and thought to play an important role in the pathophysiology of CF lung disease include the cysteine protease cathepsin B[103], cathelicidin (LL-37)[104,105], and β-defensins[106]. The activation of cathepsin B requires proteolytic removal of a prodomain, and it has been shown recently that GAGs such as CS and heparin electrostatically interact with procathepsin B and accelerate processing in a concentration-dependent manner. LL-37 demonstrates antimicrobial activity against an array of bacteria, including *S. aureus*, *Escherichia coli*[107], and *P. aeruginosa*[108], and although present in high concentrations within the CF lung, the activity of LL-37 is inhibited by binding to GAGs. On the other hand, this interaction also protects LL-37 from degradation by cathepsin D and NE, allowing concentrations of the peptide to elevate in epithelial lining fluid from individuals with CF to approximately 6.47 µM[108]. Further studies have demonstrated that NE and *Pseudomonas* LPS up-regulate the expression of β-defensin-2 in human bronchial epithelial cells[109,110]. Although binding of defensins to GAGs has been shown to inhibit the antibacterial activity of this peptide[111,112,113], GAG-defensin complexes are likely to play an important role in chemotaxis and in presenting defensins to their receptors[113].

## AIRWAY GLYCOSAMINOGLYCANS AS THERAPEUTIC TARGETS IN TREATMENT OF CYSTIC FIBROSIS

As GAG expression is altered in CF, along with strong evidence that cytokines are involved in disease pathogenesis and also interact with GAGs, this suggests that disruption of this specific interaction may be a potential adjunct to therapy impacting upon the inflammatory status of the CF lung**.** Extensive studies have been carried out in this area and specific GAG mimics have been developed to interact with chemokines[114], growth factors[115], proteases[116], and adhesion molecules[117]. *In vitro*, it has been shown that chondroitinase ABC rather than trypsin decreased viscoelasticity of CF purulent sputa[25] and in keeping with this concept, release of LL-37 within CF BALF was brought about by enzymatic digestion of GAGs (by hyaluronidase, chondroitinase ABC, or heparinase II), thereby increasing the bactericidal efficiency of CF BALF against *Pseudomonas* and *Staphylococcus* bacteria[108].

Therapies acting against airway mucus in CF include the use of hypertonic saline (HTS) (aerosolised 3–7% saline). On comparing the beneficial effects of aerosol administration of recombinant human deoxyribonuclease 1 (rhDNase) to HTS (3–7% saline)[118], results revealed that rhDNase reduced the frequency of pulmonary exacerbations[119], but illustrated a combined effect when administered with HTS for the clearance of CF purulent sputa[120]. In addition to osmotically restoring the liquid layer lining the airways[121], it has been suggested that HTS may also improve lung function by disrupting electrostatic interactions between GAGs and antimicrobial peptides. In support of this theory, *in vivo* in CF sputum, LL-37 is complexed to GAGs and is liberated following nebulised HTS, resulting in increased antimicrobial effect[108]. The results of a more recent study from our laboratory have shown that HTS decreases levels of IL-8 within CF airway secretions. The mechanism of action involved disruption of GAG:IL-8 complexes with subsequent chemokine degradation. The overall effect observed was a reduction in neutrophil chemotaxis consistent with resolution of inflammation[122]. Alternatively, rather than targeting native GAGs within the CF airways, a number of *in vitro* studies have investigated the use of polyanionic heparin as an effective mucoactive agent[123,124], which thins CF sputum by disrupting DNA-actin polymer interactions[125]. However, evidence of improved sputum clearance following inhaled heparin in CF is still lacking[126] and future evaluation involving larger doses or for a longer period of time is warranted.

## CONCLUSION

Within the lung, GAGs play a crucial role in regulating ECM structure, function, and inflammatory response. However, there is increasing evidence of dysregulated GAG expression and sulphation in the pathophysiology of CF lung disease. Whether GAG remodelling in CF is secondary to infection and inflammation or a separate process is of potential importance; if the former, then treatment of infection and inflammation could preserve normal GAG structure and function. However, if dysregulated GAG expression and sulphation are related to CFTR dysfunction, new therapeutic approaches to preserve airway function may be required. Continuing research investigating the role of GAGs in inflammation may provide a better understanding of the development and persistence of CF airway disease and ultimately may raise the fundamental question of whether these molecules, or enzymes required for their production, are targets for therapies. For example, the interaction of chemokines, cytokines, and growth factors with specific GAG sequences[57] provides a mechanism for the selection of specific inflammatory molecules at the site of inflammation. Furthermore, the specificity of this interaction and interference with chemokine/cytokine-GAG interactions could be a viable therapeutic strategy and an alternative approach to the immunologic treatment of patients with receptor antagonists and blocking antibodies. For example, blocking interactions between IFN-γ and endothelial GAGs was suggested as a clinical method of immunosuppression[127], whilst the anti-inflammatory properties of a cationic-derived peptide of mouse IFN-γ was shown to inhibit the GAG binding capacity of IL-8[128]. Such types of treatment may be useful in chronic airway inflammation to decrease concentrations of IL-8, but as these peptides are not specific, they may also interfere with binding of other inflammatory mediators. Moreover, inhibitors of *N*- and *O*-sulphotransferases resulting in reduced levels of GAG sulphation may have therapeutic relevance by altering GAG-protein interactions of pathophysiological importance. Bearing in mind the essential role of GAGs in maintaining tissue homeostasis, the actual benefits or potential risk factors associated with such treatments, and ultimately the evaluation of their possible therapeutic beneficial role in CF pulmonary disease, requires further investigation.

## ACKNOWLEDGEMENTS

We would like to thank the Medical Research Charities Group/Health Research Board, The Alpha-1 Foundation (U.S.), and the Program for Research in Third Level Institutes (PRTLI) administered by the Higher Education Authority for support.
